# Prevalence and drivers of female genital mutilation/cutting in three coastal governorates in Yemen

**DOI:** 10.1186/s12889-023-16299-y

**Published:** 2023-07-17

**Authors:** Mansour Abdu Al-Taj, Motahar Hassan Al-hadari

**Affiliations:** 1 Department of Community Medicine, Faculty of Medicine, Sana’a University, Mudbah Street, Sana’a, 773169022 Yemen; 2 Protection Programme, Human Access for Partnership and Development, Aden, Yemen

**Keywords:** Female genital mutilation/cutting, Prevalence, Risk factors, Yemen

## Abstract

**Background:**

Female genital mutilation/cutting (FGM/C), a violation of human rights, remains common in the coastal areas of Yemen.

**Objective:**

This study aimed to identify the prevalence of FGM/C and its risk factors among the youngest daughters in families in the Yemeni coastal areas, as well as the knowledge and attitudes of the local population towards FGM/C.

**Methods:**

A cross-sectional survey was conducted among 646 women and 345 men from six districts in three Yemeni coastal governorates between July and September 2020 using a structured questionnaire. Categorical data were described by proportion. The chi-square test was used to identify factors associated with FGM/C. All factors with a p-value of ≤ 0.05 were included in the multivariate analysis. Adjusted odds ratios (AORs) and 95% confidence intervals (CIs) were calculated in the multivariate logistic regression analysis.

**Results:**

The prevalence of FGM/C in Yemeni coastal areas was 89.0% (95% CI 84.0%-92.5%) among women and 79.8% (95% CI 73.5%-84.8%) among the youngest daughters in the surveyed families. Nearly two-thirds of women and half of the men recorded a poor knowledge level about the harms of FGM/C. Furthermore, almost two-thirds of both women and men would like to continue the practice of FGM/C. Among women, significant predictors of FGM/C among youngest daughters included advanced maternal age of ≥ 40 years (AOR 7.16, 95% CI 2.73–18.76), mother’s desire to continue FGM/C (AOR 8.07, 95% CI 3.64–17.89), and living in a rural area (AOR 3.95, 95% CI 1.51–10.30). Daughters of mothers who did not undergo FGM/C were more protected from FGM/C than those whose mothers had undergone FGM/C (AOR 0.04, 95% CI 0.02–0.09). Among men, the father’s desire to continue FGM/C (AOR 15.10, 95% CI 6.06–37.58) was significantly associated with FGM/C among the youngest daughters.

**Conclusion:**

This study confirmed that FGM/C is still prevalent among communities in Yemeni coastal areas. Thus, community-based interventions with a focus on the rural population are vital to improving the awareness of various harms of FGM/C.

## Introduction

Female genital mutilation/cutting (FGM/C) is a form of violence against women that entails partial or complete removal of their external genitalia. It is widely practised in Africa and Asia [1–3]. FGM/C can expose the affected women to multiple lifelong health problems during the time of removal, puberty, marriage, and pregnancy. Some of the immediate consequences of FGM/C are pain, bleeding, infection, shock [3, 4], and even death [5]. In the long term, FGM/C exposes women to psychological problems [6–8] such as depression, anxiety, and post-traumatic stress disorder [9]. In addition, women who have undergone FGM/C are prone to sexual dysfunction as it may decrease their sexual desires [10, 11]. Moreover, FGM/C is associated with obstetric complications [12–15]. Globally, FGM/C costs the health system approximately $1.4 billion annually [16, 17]. Despite all these implications, FGM/C is still practised widely in certain parts of the world. According to the latest statistics, more than 200 million women and girls in 30 countries in Africa, the Middle East, and Asia have undergone FGM/C [16].

There are four types of FGM/C. Type 1 is the partial or total removal of the glans of the clitoris. Type 2 is the partial or total removal of the glans clitoris and the labia minora, with or without removal of the labia majora. Type 3, known as suturing, is the narrowing of the vaginal opening. Lastly, type 4 includes all other harmful procedures involving the female genitals for non-medical purposes, such as pricking, piercing, incision, scraping, and cauterisation [16]. FGM/C is usually performed by traditional practitioners. In rare instances in some areas, it is also conducted by health workers [3, 18]. Most FGM/C is performed with non-sterile instruments, thus often leading to infection [3, 18]. Custom, tradition, religion, and female sexuality are the historical reasons behind the practice of FGM/C in some families and communities [18]. Apart from that, recent studies have identified several risk factors of the practice, including low maternal education [19–21], older mothers [19], poor family economic status [19], residency in rural areas [19, 20], and a maternal history of FGM/C [19, 21].

Situated in the Arab Peninsula, Yemen has been plagued by a series of wars, making it a country that suffers some of the worst humanitarian crises in the world, accompanied by a high rate of violence against women [22]. The overall prevalence of FGM/C in Yemen is 19% among women aged 15 to 49 years. However, it is more prevalent in the coastal regions, namely Al-Mahrah (84.4%), Hadramout (79.9%), and Al-Hodeidah (62.3%) [23]. In comparison, the prevalence of FGM/C in the rest of the governorates is lower, ranging from 0% in Al-Baidha to 21.5% in Reimah [23]. About 85% of FGM/C among girls was performed during the first week after birth, followed by approximately 15% in less than a year [23]. According to a survey in 2008–2009, older women, low household income, and low maternal education were associated with FGM/C [24]. Moreover, a secondary analysis of Yemen’s 1997 and 2003 demographic health surveys showed that women who had undergone FGM/C and their husbands were more likely to support the continuation of the practice among their daughters compared to those who did not undergo FGM/C [25].

To date, no law or regulation in Yemen prohibits the practice of FGM/C. In 2008, a Safe Motherhood bill was submitted to the Yemeni Parliament, but it was subsequently rejected due to a clause prohibiting FGM/C [25]. Continuous efforts have been made to reduce this practice. Many local organisations, supported by international partners such as the United Nations (UN), have implemented projects to reduce gender-based violence (GBV), including FGM/C in Yemen’s coastal governorates. To obtain baseline data on the behaviours, attitudes, and risk factors associated with GBV in Yemeni coastal areas, a survey was conducted by the Human Access for Partnership and Development with the support of the UN Fund for Population Activities. One of the main components of the survey revolved around the prevalence and risk factors of FGM/C among women and girls, the focus of our present study. The findings will serve as important guidelines for policy makers and international donors in the decision-making pertaining to the priorities and impact of ongoing and future projects aiming to mitigate FGM/C.

## Methods

### Study design

A cross-sectional survey was conducted between July and September 2020 in the Hadramout, Al-Mahrah, and Al-Hodeidah governorates in Yemen.

### Study population and setting

The study population included women and men in the six districts within the three governorates. The targeted districts were Seiyun, Mukalla, and Ash Shihr in Hadramout; Hays and Al Khawkha in Al-Hodeidah; and Al Ghaydah in Al-Mahrah. This study was part of the GBV Protection Project implemented by the Human Access for Partnership and Development.

### Sample size

A multiple indicator cluster survey strategy developed by UNICEF was used to calculate the required sample size. Using a 20% prevalence of violence among women and men based on a previous study [26], the sample size was obtained with two design effects based on a proportion of women and men in the total population of 0.5, a 0.12 relative margin of error at a 95% CI, an average family size of six members per household, a cluster size of 20 households, and a 20% nonresponse rate, giving rise to the required sample size of 926 households. The number of households was further increased to 1,000 and divided by 20, resulting in 50 clusters. As women experience different types of GBV at a higher frequency compared to men, the strategy was designed to target almost two-thirds of households with women (n = 650) and one-third of those with men (n = 350).

### Sampling

A multistage cluster sampling was used. Each district was first divided into enumeration areas according to the 2004 census data from the Central Statistical Organisation. The sample size for each district was then determined proportionally according to population size based on the UN Office for the Coordination of Humanitarian Affairs projection for the year 2019 [27]. Enumeration areas were then selected for each district via simple random sampling. Lastly, households within those enumeration areas were selected using the method implemented by the World Health Organization Expanded Program on Immunisation surveys for rural and urban areas [28]. In each enumeration area, 13 houses (one woman from each house) and seven houses (one man from each house) were randomly selected. If there was more than one family in the same house, only one family would be chosen at random.

### Data collection

Face-to-face interviews with women and men were carried out using structured questionnaires in their homes or places that ensured privacy and confidentiality. If the mothers were not available due to death, travel, or inability to remember, the eldest daughter who was the household’s primary caregiver would be interviewed instead. Similarly, if the fathers were unavailable on the interview days, the eldest son would be interviewed. The questionnaire contained different sections that captured data related to GBV, including FGM/C practice, knowledge, and attitude, as well as the characteristics of households, parents, and heads of households. The interviews were conducted by nine teams, each consisting of three women, a man, and a supervisor. All the team members received two days of training on data collection and questioning methods. On the third day, a pilot study was conducted before the actual data collection.

### Study variables

The outcome variable was the practice of FGM/C. A woman was considered to have had FGM/C if she reported the removal of any part of external genitalia for non-medical reasons. A daughter was considered to have had FGM/C if her mother or father reported the removal of any part of her external genitalia for non-medical reasons [3]. To measure the prevalence of FGM/C among youngest daughters, the respondents were asked whether they had at least one daughter and if she had undergone FGM/C. The prevalence of FGM/C among youngest daughters was calculated as the total number of youngest daughters who had undergone FGM/C divided by 771 (the total number of households with at least one daughter).

Using 11 questions about the respondent’s knowledge of the physical, psychological, and social harms of FGM/C, an index was established to indicate the knowledge as either good or poor. A respondent’s attitude toward FGM/C was measured by their future desires to continue or stop the practice. For those who stated the desire to continue, potential reasons were enquired based on the five categories of virginity preservation, purity, societal acceptance, for religious requirement, and improved marriage prospects. The person who performed FGM/C could be a healthcare worker or a traditional practitioner. Other sociodemographic characteristics of the respondents that were obtained included their age in years (˂30, 30–39, and ≥ 40), education level (no education, primary, and secondary level and above), employment status (employed and unemployed), and place of residence (urban and rural).

### Statistical analysis

Stata 16 (Stata Corp., College Station, Texas, USA) was used to analyse the data. Missing values were excluded. To adjust for the unequal probability of selection among the clusters, data were weighted using the “svy” command. Descriptive statistics were used to present the variables. To reduce the number of items under the knowledge category, principal components analysis with varimax rotation was used. A predictive value was then generated based on the median score. Scores above the median value indicate good knowledge while scores below the median indicate poor knowledge. The internal consistency was assessed using Cronbach’s alpha, which yielded a value of 0.794, indicating good consistency.

To obtain an updated understanding of the risk factors of FGM/C, bivariate analysis (Chi-square test) was performed to determine the risk factors associated with FGM/C among youngest daughters. Only variables with p-values of ≤ 0.05 were included in the multivariate logistic regression, except for the mother’s education which was included prior. In the bivariate analysis for the men’s group, the association between the sex of the head of the household and FGM/C among the youngest daughters was significant. However, one cell was zero. Thus, the variable of the gender of the household head was not included in the multivariate analysis.

## Results

Four of the 650 women and five of the 350 men refused to participate in the study, resulting in a final sample size of 646 women and 345 men. Approximately 20.0% of women (n = 138) and men (n = 65) were younger than 30 years old. Most of them were of a low education level as only less than 20.0% (n = 105) of women and 45.0% (n = 150) of men had secondary education or higher. The majority of women (94.4%, n = 607) and two-thirds of men (67.8%, n = 232) were not employed. In terms of marital status, around 90.0% of women (n = 602) and men (n = 307) were married or have been married. Generally, most of the households (n = 931) were headed by men and almost half of the households (n = 497) stayed in rural areas (Table 1).

**Table 1:** Characteristics of respondents

	Total		Women N = 646				Men N = 345	
Variable	n	%		n	%	n		%
Respondent’s Age (years)								
<30	203	20.9		138	22.0	65		19.0
30–39	303	31.2		213	34.0	90		26.2
≥ 40	464	47.8		276	44.0	188		54.8
Respondent’s education level								
No education	563	56.9		443	68.6	120		35.0
Primary	171	17.3		98	15.2	73		21.3
Secondary and above	255	25.8		105	16.2	150		43.7
Respondent’s employment status								
Employed	146	14.8		36	5.6	110		32.2
Not Employed	839	85.2		607	94.4	232		67.8
Marital status								
Ever Married	909	91.8		602	93.3	307		89.0
Single	81	8.2		43	6.7	38		11.0
Sex of the head of household								
Male	931	94.0		590	91.3	341		99.1
Female	59	6.0		56	8.7	3		0.9
Family had at least one daughter								
Yes	776	86.7		515	86.7	261		86.7
No	119	13.3		79	13.3	40		13.3
Place of residence								
Urban	494	49.8		324	50.2	170		49.3
Rural	497	50.2		322	49.8	175		50.7

N, sample size; n, number; %, percentage

Table 2 presents the prevalence, knowledge, and attitudes of FGM/C among women and girls. The prevalence of FGM/C was 89.0% (95% CI 84.0%-92.5%) and 79.8% (95% CI 73.5%-84.8%) among women (n = 572) and youngest daughters (n = 615) respectively. Among households with daughters (n = 771), 84.6% (95% CI 79.0%-88.9%) of households (n = 652) had at least one daughter who had undergone FGM/C. Moreover, 46.5% (n = 282) of FGM/C among youngest daughters was performed by traditional practitioners. In terms of knowledge and attitude, approximately two-thirds of women (70.8%, n = 449) and half of all men (48.8%, n = 168) reported poor knowledge about the harmful effects of FGM/C and 70.0% of women (n = 441) and men (n = 234) expressed a desire to subject their daughters to FGM/C in the future. Women indicated their desire to continue practising FGM/C among their daughters was driven by the promotion of girls’ cleanliness (65.8%, n = 289), for religious requirement (46.0%, n = 202), virginity preservation (26.6%, n = 117), and to improve the marriage prospects (1.6%, n = 7). Similarly, men also quoted similar reasons, i.e. promoting girls’ cleanliness (65.4%, n = 153), for religious requirement (43.6%, n = 102), virginity preservation (39.3%, n = 92), and improved marriage prospects (11.5%, n = 27) as the reasons behind their support for FGM/C. Among those who were against the continuation of FGM/C, almost half of women (47.3%, n = 89) and men (50.5%, n = 54) considered it as a bad habit, and one-quarter of them felt that it was against the religion. Another 39.3% (n = 42) of men and 30.3% (n = 57) of women perceived FGM/C as violence against women (Table 2).

**Table 2:** Prevalence, knowledge, and attitudes of respondents on female genital mutilation

	Total		Women		Men	
Variable	n	*%*	n	*%*	n	*%*
Women have undergone FGM/C^#^ (N = 643)						
Yes			572	89.0		
No			71	11.0		
At least one daughter in household has undergone FGM/C (N = 771)						
Yes	652	84.6	446	86.6	206	80.5
No	119	15.4	69	13.4	50	19.5
Youngest daughter has undergone FGM/C (N = 771)						
Yes	615	79.8	425	82.5	190	74.2
No	156	20.2	90	17.5	66	25.8
Person who performed FGM/C on youngest daughter (N = 607)						
Traditional practitioner	282	46.5	200	47.2	82	44.8
Health workers	325	53.5	224	52.8	101	55.2
Respondent’s knowledge of FGM/C (N = 978)						
Good	361	36.9	185	29.2	176	51.2
Poor	617	63.1	449	70.8	168	48.8
Respondent’s opinions on continuing to practice FGM/C (N = 987)						
Continue	675	68.4	441	68.5	234	68.2
Discontinue	312	31.6	203	31.5	109	31.8
Reasons for continuation						
Cleanliness for girls	442	65.7	289	65.8	153	65.4
Social desire	121	18.0	50	11.4	71	30.3
Ensure girls’ virginity	209	31.1	117	26.6	92	39.3
For Religious requirement	304	45.2	202	46.0	102	43.6
Improved marriage prospects	34	5.1	7	1.6	27	11.5
Reasons for discontinuation (N = 295)						
Bad habit	143	48.5	89	47.3	54	50.5
Against religion	78	26.4	50	26.6	28	26.2
Cause medical complication	98	33.2	49	26.2	49	45.8
Painful experience	61	20.7	40	21.3	21	19.6
Violence against women	99	33.6	57	30.3	42	39.3

N, sample size; n, number; %, percentage; Women have undergone FGM/C#, mother, or eldest daughter

**Table 3:** Unadjusted odds ratios for the FGM/C risk factors among youngest daughters by men and women

	Women (N = 515)			Men (N = 256)		
	FGM/C among youngest daughter					
Variable	n	%	P-value	n	%	P-value
Respondent’s Age (years)			< 0.001			< 0.001
<30	43	65.2		12	44.4	
30–39	142	78.9		42	68.8	
≥40	225	88.6		135	81.3	
Respondent’s education level			0.381			0.051
No education	319	83.7		77	77.8	
Primary	59	81.9		49	83.0	
Secondary and above	47	75.8		64	66.7	
Respondent’s employment status			0.151			0.305
Employed	13	68.4		69	78.4	
Not Employed	411	83.0		121	72.9	
Mother has undergone FGM/C			< 0.001			
Yes	414	89.2				
No	9	18.7				
Respondent’s knowledge of FGM/C			0.001			0.080
Good	106	74.1		94	68.6	
Poor	309	85.6		96	80.7	
Respondent’s opinions on continuing to practice FGM/C			< 0.001			< 0.001
Discontinue	73	51.1		32	40.0	
Continue	350	94.6		158	89.8	
Sex of the head of household			0.339			0.011
Male	389	82.1		190	75.1	
Female	36	87.8		0	0.0	
Place of residence			0.001			0.126
Urban	181	72.4		87	68.5	
Rural	244	92.1		103	79.8	
Governorate			< 0.001			< 0.001
Hadramout	359	88.4		153	80.5	
Al-Hodeidah	46	69.7		28	60.9	
Al-Mahrah	20	46.5		9	45.0	

N, sample size; n, number; %, percentage; FGM/C, female genital mutilation/Cutting

Table 3 shows the characteristics of respondents by the youngest daughter’s FGM/C status. Among women, the prevalence of FGM/C among the youngest daughters was higher with an increase in maternal age (P < 0.001). On the other hand, the prevalence of FGM/C among the youngest daughters decreased from 83.7% among those with no formal education to 75.8% among those with a secondary or higher level of education. However, this difference was not statistically significant (P = 0.381). The proportion of FGM/C was higher among youngest daughters whose mothers had experienced FGM/C than those who did not undergo FGM/C (P < 0.001), as well as among those whose mothers had poor knowledge about the harms of FGM/C compared to those who were better informed (P = 0.001). In addition, the prevalence of FGM/C was statistically higher if the mother expressed the desire to continue the practice of FGM/C (P < 0.001). Those living in rural areas also recorded a higher prevalence of FGM/C among their youngest daughters than those living in urban areas (P = 0.001).

As for men, the prevalence of FGM/C among the youngest daughters increased from 44.4% if the father was less than 30 years old to 81.3% if the father was 40 years or older (P < 0.001). The prevalence was higher among those whose fathers had primary education (83.0%) than those with no education (77.8%) or secondary education and higher (66.7%). Furthermore, daughters whose fathers expressed a desire to continue the practice of FGM/C reported a higher prevalence of FGM/C among their youngest daughters compared to their counterparts (Table 3). Additionally, for both male and women, the prevalence of FGM/C was higher in Hadramout compared to Al-Hodeidah and Al-Mahrah (Table 3).

In the multivariate analysis involving women, the odds of FGM/C were 7.16 times higher among the youngest daughters whose mothers’ age were ≥ 40 years (AOR 7.16, 95% CI 2.73–18.76) than those younger than 30 years old. In addition, the odds of FGM/C were eight times higher among the youngest daughters whose mothers support the continuation of FGM/C (AOR 8.07, 95% CI 3.64–17.89). Furthermore, those whose mothers did not undergo FGM/C before were less likely to have FGM/C (AOR 0.04, 95% CI 0.02-0.09). In terms of residence, those living in rural areas were at 3.95 times higher risk of being subjected to FGM/C (AOR 3.95, 95% CI 1.51–10.30) while those residing in Al-Hodeidah (AOR 0.30, 95% CI 0.11–0.82) and Al-Mahrah (AOR 0.24, 95% CI 0.11–0.53) governorates were less likely to have FGM/C compared with those residing in Hadramout. Lastly, mothers ’education and mothers’ knowledge were not significantly associated with FGM/C among the youngest daughters. As for men, the significant predictor of FGM/C among youngest daughters was fathers who supported the continuation of FGM/C (AOR 15.10, 95% CI 6.06–37.58) (Table 4).

**Table 4:** Multivariate analysis for the FGM/C risk factors among the youngest daughter in the coastal areas of Yemen

Variable	Women		Men	
	AOR (95% CI)	p-value	AOR (95% CI)	p-value
Respondent’s Age (years)				
<30	Ref.		Ref.	
30–39	2.12 (0.67 − 6.73)	0.198	0.83 (0.26–2.72)	0.758
≥ 40	7.16 (2.73–18.76)	< 0.001	1.45 (0.52– 4.06)	0.466
Respondent’s education level				
No education	Ref.		Ref.	
Primary	1.49 (0.63 − 3.55)	0.361	0.67 (0.22–2.01)	0.466
Secondary and above	0.99 (0.42 − 2.35)	0.990	0.63 (0.28–1.41)	0.255
Respondent’s knowledge of FGM/C				
Good	Ref.			
Poor	1.02 (0.47 − 2.20)	0.960		
Respondent’s opinions on continuing to practice FGM/C				
Discontinue	Ref.		Ref.	
Continue	8.07 (3.64 − 17.89)	< 0.001	15.10 (6.06–37.58)	< 0.001
Mother has undergone FGM/C				
Yes	Ref.			
No	0.04 (0.02 − 0.09)	< 0.001		
Place of residence				
Urban	Ref.			
Rural	3.95 (1.51 − 10.30)	0.006		
Governorate				
Hadramout	Ref.		Ref.	
Al-Hodeidah	0.30 (0.11–0.82)	0.019	0.54 (0.27–1.07)	0.077
Al-Mahrah	0.24 (0.11–0.53)	0.001	0.10 (0.01 − 0.74)	0.025

AOR, Adjusted Odds Ratios; 95% CI, 95% of confidence intervals; FGM/C, female genital mutilation/Cutting; Ref., reference group

## Discussion

This study was conducted among households in three governorates of Yemen where FGM/C was still commonly practised. As part of the study’s aim to identify intergenerational trends, the FGM/C prevalence among women and their youngest daughters was determined, along with the identification of risk factors associated with FGM/C among the youngest daughters. The prevalence of FGM/C among women obtained in this study (89.0%) differed widely from the prevalence obtained in a national survey (19.0%) [23]. One of the possible reasons could be the catchment area as the national survey included governorates known to have a low prevalence of FGM/C whereas our study focused on the coastal areas where FGM/C is still widely practised in the community.

In comparison with other countries, the prevalence of FGM/C in this study was close to that in Sudan (87.2%) [29], Egypt (90.0%) [30], and slightly lower than that in Djibouti (95.5%) [31] and Somalia (99.2%) [32]. However, it was considerably higher than other Arab countries, such as 18.2% in Saudi Arabia [33], 41.4% in the United Arab Emirates (UAE) [34], and 50.1% in the Erbil governorate of Iraq [35]. The difference in the prevalence rate was most likely due to the purposive selection of regions with high rates of FGM/C as study sites in our study. Apart from that, Iraqi and Emirati women also reported a higher level of education which might have an impact on the lower rates of FGM/C.

When compared to mothers, the prevalence of FGM/C among the youngest daughters was slightly lower by approximately 9%. This cross-generational gap may be a good indication of the attempts to combat the practice of FGM/C in these societies via the collaboration between the Yemeni government and international partners. However, more awareness programmes to deter the practice of FGM/C are still needed to further reduce the prevalence. The prevalence of FGM/C among the youngest daughters in this study was higher than those reported in the UAE [34], Ethiopia [36], and Sudan [37]. As abovementioned, we postulated that the differences in the prevalence were attributed to the different study populations, i.e. the Ethiopian and Sudanese studies were secondary analyses of health and demographic surveys that led to a prevalence representative of an entire country as compared to our findings that focused on highest groups at risk in certain regions.

In addition, this study reported that it was more common for traditional practitioners to perform FGM/C. This was in contrast with the findings from an Ethiopian study in which 94.4% of FGM/C among the surveyed daughters was performed by traditional practitioners [38]. Despite the high risk of infection from the use of contaminated tools by traditional practitioners, many families continue to request their services due to their faith and conviction in their expertise, as well as their trust in the traditional practitioners to keep the FGM/C confidential.

Nevertheless, more than half of the FGM/C of youngest daughters in this study was performed by healthcare staff despite a ministerial decree prohibiting the practice of FGM/C in both governmental and private health facilities [25]. In comparison, 73.7% of FGM/C on youngest daughters in Emirati households was performed by healthcare workers, likely because more households can afford the charges at healthcare facilities compared to Yemeni households [34]. Having FGM/C performed by qualified healthcare staff may reduce the risk of infection and distortion to the female external reproductive system. A recent review found that the main motivating factors for healthcare workers to perform FGM/C were reduced harm to girls, cultural reasons, financial gains, and responding to community requests [39]. On the other hand, legalising FGM/C or allowing healthcare staff to perform the procedure without a legal justification may wrongly foster the faith of the community members that FGM/C is a safe procedure, thereby potentially increasing its prevalence. As more than half of the FGM/C in this study was performed by healthcare workers, the policy makers need to re-evaluate if the practice should be banned altogether or to develop a safety guideline that enables the healthcare workers to perform FGM/C before it can be gradually eliminated with the proper awareness and educational campaigns.

Similar to previous reports, women’s intention to continue the practice of FGM/C for their daughters was statistically related to the presence of FGM/C among existing daughters [40]. The desire to continue FGM/C shown by the women and men in our study is likely influenced by the community and religious norms that promote the practice. Yemeni societies are made up of tribal systems governed by customs and traditions and almost all women and men follow these customs and traditions. In certain areas, FGM/C is commonly practised as it is an unwritten social convention [16]. The fear of social exclusion propels many people to follow their fellow community members and take up FGM/C practice [16]. Religious beliefs also support these social norms. Reports have indicated that religious beliefs are linked to the occurrence of FGM/C [21, 41]. In contrast, findings from a secondary analysis in Kenya highlighted that girls living in a community that discouraged FGM/C were less likely to have FGM/C [42].

Our results are consistent with findings from Kenya [43] and Chad [44] whereby mothers who had undergone FGM/C were more likely to undergo FGM/C. This is contrary to the expected belief that mothers who had undergone FGM/C would be less likely to subject their daughters to FGM/C as a result of the psychological and physical harm they experienced. One of the reasons could be the lack of influence of women’s opinion in the household as men are usually the decision-makers due to the societal norms and traditions in Eastern cultures such as Yemeni societies.

With regard to the urban-rural difference, the prevalence of FGM/C among the daughters of women was higher in the rural areas while the difference was not significant among the men. Many factors could explain the higher prevalence of FGM/C in rural areas. For instance, Yemeni villages usually consist of a small number of tribally-cohesive families that practised insular customs and traditions. In contrast, urban cities are often inhabited by families from different places with various customs and traditions. Furthermore, as a result of the lack of electricity, there is a low penetration of media and the Internet among rural residents who are mostly agricultural farmers. Last but not least, illiteracy among women, men, boys, and girls in rural areas could have also contributed to the low knowledge and high prevalence of FGM/C.

In women, the age of the mother being 40 years and above was statistically linked to FGM/C among youngest daughters. This explains that the practice of FGM/C may decrease in the younger generations. Unexpectedly, and in contrast to previous studies in Chad [44] and Sudan [45], the mothers’ education was not statistically associated with the FGM/C of the youngest daughters in our study. The lack of association between maternal education and FGM/C among youngest daughter might have been mediated by other factors such as social and cultural norms that were not evaluated in this study. Although education is a powerful tool in promoting positive changes and reducing harmful practices such as FGM/C, it can often take a long time for behavioural modifications, especially in countries or regions where societal pressure is strong [46]. Furthermore, the bivariate analysis showed that poor knowledge of mothers about the harms of FGM/C was associated with FGM/C among youngest daughters, but this relationship was not significant following multivariate regression. One of the possible reasons might be the strong influence of the respondent’s beliefs on the continuation of FGM/C. A recent meta-analysis revealed that despite an increased level of awareness, positive attitudes toward the eradication of FGM/C remained low in many counties [47].

Last but not least, the prevalence of FGM/C among the youngest daughters of women in Al-Hodeidah and Al-Mahrah was lower than in Hadramout. Similarly, the youngest daughters of men in Al-Mahrah were less likely to be subjected to FGM/C than those in Hadramout. The differences between these two areas may be related to their customs and traditions. Likewise, Al-Hodeidah is considered to be more vulnerable among all the provinces. For the past 10 years, international and local organisations have been actively organising various women’s protection programmes in Al-Hodeidah, which may have contributed to a reduced prevalence of FGM/C. Variations in the prevalence of FGM/C between regions in the same country have been reported in different contexts [48, 49]. In the recent national survey, Al-Mahrah recorded the highest prevalence of FGM/C in Yemen [23]. The lower prevalence in Al-Mahrah than in Hadramout recorded in this study was possibly due to the internal displacement caused by the civil war. Although this study did not address any factors related to war, a post-study follow-up survey revealed that most of those who had not undergone FGM/C were families displaced from the conflict areas in northern Yemen where the practice is less common.

### Limitations

The study faced some limitations and challenges. Efforts to minimise non-response and other potential biases were attempted by collecting data in a place with guaranteed privacy. However, some participants were reluctant to answer certain sensitive questions during the pilot study. Thus, the question about the type of FGM/C was removed from the questionnaire. In addition, there was also potential recall bias for questions asked about past events. Furthermore, the sample size was initially calculated for a wider study on GBV. Thus, the sample size might be inadequate for the assessment of certain statistical associations in the multivariate model, for example, the difference between urban and rural men in relation to the practice. Lastly, this study also did not capture important demographic characteristics of the youngest daughters, the age when FGM/C was performed, and other war-related factors.

Despite the limitations, there are certain study strengths. Data obtained from 50 clusters or villages with a wide range of practices and beliefs meant that the results from this study are considered generalisable to the coastal areas in Yemen. Furthermore, the availability of trained data collectors who were given sufficient time to conduct interviews increased the accuracy and quality of the results.

## Conclusion

FGM/C is still a common practice in the coastal areas of Yemen. Mothers’ and fathers’ strong beliefs about FGM/C, advanced maternal age of 40 years and above, rural residence, and mothers’ history of FGM/C were the strongest predictors of FGM/C among the current generation of girls. Interventions targeting different community sectors, such as women and men, religious men, rural societies, male and female students, as well as health workers, are vital to correct the misconceptions surrounding FGM/C.

## Data Availability

The data that support the findings of this study are included in the manuscript. Datasets are available from the corresponding author upon reasonable request and with permission of [Human Access for Partnership and Development].

## Abbreviations

FGM/C: Female genital mutilation/Cutting
AOR: Adjusted odds ratio
CI: Confident intervals
GBV: Gender-based violence
UN: United Nations
UAE: United Arab Emirates
